# Impact of suppressing ciprofloxacin susceptibility results on antibiotic utilization and hospital-acquired *Clostridioides difficile* infection

**DOI:** 10.1017/ash.2021.178

**Published:** 2021-08-05

**Authors:** Bryan P. White, Daniel B. Chastain, Karen K. Kinney, Katie Thompson, Jerry Kelley, Cindy B. McCloskey

**Affiliations:** 1 Oklahoma University Medical Center, Oklahoma City, Oklahoma; 2 University of Oklahoma College of Medicine, Oklahoma City, Oklahoma; 3 University of Georgia College of Pharmacy, Albany, Georgia

Fluoroquinolones are broad-spectrum antibiotics associated with multiple adverse effects and an increased risk of *Clostridioides difficile* infection (CDI).^1^ Previous data suggest that suppressing fluoroquinolone results on antimicrobial susceptibility reports leads to decreased fluoroquinolone use and increased susceptibility to *Escherichia coli*.^2^ Cascade and selective reporting of antimicrobial susceptibility results are recommended by Infectious Diseases Society of America (IDSA) stewardship guidelines.^3^ Internal data have shown that ciprofloxacin was commonly initiated for empiric therapy for urinary tract infection (UTI) and was not de-escalated. However, the effect of suppressing fluoroquinolone susceptibility results on rates of CDI^2^ has not been examined. In this study, we examined the impact of suppressing ciprofloxacin susceptibility results on antibiotic use, antibiotic susceptibility, and rates of CDI.

## Methods

This single-center quasi-experimental study at an inpatient academic medical center was conducted to determine the effect of suppressing ciprofloxacin susceptibility results from hospitalized patients. Ciprofloxacin susceptibility was the only fluoroquinolone previously reported on pansusceptible urine isolates of *Klebsiella* spp and *Escherichia coli*, including ampicillin for *E. coli*. The study was reviewed and approved by the Institutional Review Board of the Oklahoma University Medical Center. The study was performed over two 11-month periods, before the intervention began in March 2018 (April 2017–February 2018) and after the intervention (April 2018–February 2019). The suppression was done automatically by the microbiology laboratory, and results were made available to providers upon telephonic request. The study was conducted at a hospital with robust stewardship strategies including audit and feedback, prior authorization and restriction, and rapid diagnostics, but no restrictions on fluoroquinolone use. The primary objective was to compare the utilization of levofloxacin and ciprofloxacin in adults before and after the intervention. Secondary objectives included monthly antibiotic utilization, defined as days of therapy per 1,000 patient days (DOT/1,000 PD) for levofloxacin, ciprofloxacin, ceftriaxone, trimethoprim/sulfamethoxazole (TMP/SMZ), fosfomycin, cephalexin, and nitrofurantoin. Other secondary objectives were hospital-acquired CDI (HA-CDI) rates as defined by the Centers for Disease Control and Prevention (CDC) and *Pseudomonas aeruginosa* susceptibility to ciprofloxacin isolated from any culture. At our medical center, CDI is diagnosed with a 1-step polymerase chain reaction (PCR) assay, and this protocol did not change after the intervention. Pre-2019 Clinical and Laboratory Standards Institute (CLSI) break points for *P. aeruginosa* and fluoroquinolone were used throughout the study.^4^ An interrupted time series analysis using Stata MP version 12.1 software (StataCorp, College Station, TX) was conducted to assess the intercept (level) and slope (rate) of a trend line before and after the intervention. Drug utilization reports for ciprofloxacin, levofloxacin, ceftriaxone, TMP/SMZ, fosfomycin, cephalexin, and nitrofurantoin, as well as reports for *P*. *aeruginosa* susceptibility to ciprofloxacin, were obtained on a monthly basis from VigiLanz, a clinical decision support system.

## Results

No change in the intercept or slope of ciprofloxacin DOT/1,000 PD (0.27, 95% CI: −0.94 to 1.48 vs 3.49; 95% CI, −10.89 to 3.90) or levofloxacin DOT/1,000 PD (−5.87, 95% CI, −17.79 vs 6.06; −0.98, 95% CI, −2.86 to 0.90) occurred after the intervention (Table 1). The absolute DOT/1,000 PD of levofloxacin and ciprofloxacin before and after the intervention were 121.46 and 95.38, respectively. The ceftriaxone DOT/1,000 PD intercept decreased after the intervention (*P* = .01), but the slope did not change. The cephalexin and nitrofurantoin DOT/1,000 PD intercepts (*P* = .01 for both) increased after the intervention without changes in their slopes. We detected no change in the intercept or slope of fosfomycin DOTs/1,000 PD or TMP/SMZ DOTs/1,000 PD or in the slope of HA-CDI (Table 1). In total, 820 *P. aeruginosa* cultures were included in the analysis, with a median of 38.5 cultures per month (IQR, 31–42). Also, 1 month before the intervention and 2 months after the intervention, we detected <30 *P. aeruginosa* isolates (range, 24–28). The intercept of *P*. *aeruginosa* susceptibility to ciprofloxacin exhibited an increasing trend (8.13; 95% CI, 0.00–16.26), and the slope increased after the intervention (1.65; 95% CI, 0.44–2.87).

**Table 1.** Presuppression and Postsuppression Rate Changes

Variable	Slope Change (95% CI)	*P* Value	Intercept Changes (95% CI)	*P* Value
Levofloxacin DOT/1,000 PD	−0.98 (−2.86 to 0.90)	.29	−5.87 (−17.79 to 6.06)	.32
Ciprofloxacin DOT/1,000 PD	0.27 (−0.94 to 1.48)	.64	−3.49 (−10.89 to 3.90)	.33
Hospital-acquired CDI	−0.109 (−0.983 to 0.766)	.80	4.1 (0.431–8.631)	.073
Ceftriaxone DOT/1,000 PD	0.78 (−0.36 to 1.91)	.17	−12.62 (22.13 to −3.11)	.01
TMP/SMZ DOT/1,000 PD	−0.13 (−1.10 to 0.84)	.78	−3.61 (−9.21 to 1.99)	.19
Fosfomycin DOT/1,000 PD	−0.004 (−0.68 to 0.11)	.14	−0.29 (−0.68 to 0.11)	.14
Cephalexin DOT/1,000 PD	−0.15 (−0.52 to 0.21)	.38	2.71 (0.86–4.57)	.01
Nitrofurantoin DOT/1,000 PD	0.17 (1.03–5.90)	.45	3.47 (1.03–5.90)	.01
*P. aeruginosa* ciprofloxacin susceptibility	1.65 (0.44–2.87)	.01	8.13 (0.00–16.26)	.05

Note. DOT, days of therapy; PD, patient days; TMP/SMZ, trimethoprim/sulfamethoxazole.

## Discussion

Selective reporting of susceptibility results on urinary cultures has been shown to change prescribing practices in inpatient and outpatient settings.^2,6^ Although recommended in the guidelines, a recent survey of 36 European countries showed that 58% have not adopted selective reporting.^3,7^ Barriers to selective reporting included lack of implementation guidelines, other priorities, and lack of human and information technology resources.^7^ In contrast, a 2017 survey of 94 microbiology laboratories in New Zealand and Australia reported that 87% of laboratories suppressed ciprofloxacin susceptibility results from pansusceptible *E*. *coli* urinary cultures.^10^ With low stewardship staffing levels, antimicrobial stewardship programs should consider using selective reporting more aggressively. Furthermore, antimicrobial stewardship staffing ratios of up to 1 pharmacist per 100 occupied beds have been suggested.^8^ A recent survey of 78 top US hospitals, as ranked by *US News and World Report*, showed that 62% of hospitals had 1 pharmacist full-time equivalent or less devoted to antimicrobial stewardship activities.^9^ Suppressing ciprofloxacin susceptibility results on pansusceptible *Klebsiella* spp and *E. coli* isolated from urine cultures was associated with an increased slope of *P. aeruginosa* susceptibility to ciprofloxacin and increased cephalexin and nitrofurantoin DOTs. No changes were seen in fluoroquinolone DOTs/1,000 PD or HA-CDI rates. Stewardship programs should consider implementing selective reporting in combination with 1 or more other initiatives based on local challenges and workload to maximize the approach of microbiology nudging to influence prescribing.

This study has several limitations. We did not collect antibiotic indications, which would have allowed us to determine the percentage of fluoroquinolones used for UTI that was affected by the intervention. Prior to implementing the intervention, fluoroquinolone warnings were announced hospital-wide, which may have contributed to lower DOTs. As the fluoroquinolone DOTs/1,000 PD did not change, changes in *P*. *aeruginosa* susceptibility may have been due to a short duration of follow-up, as well as other unrecognized interventions or changes. Additionally, in 3 study months there were <30 *P. aeruginosa* isolates, and the CLSI recommends at least 30 isolates for antibiogram creation.^5^ Discharge prescription data were not collected, but they will be the focus of a future study.

## References

[1] Leffler DA , Lamont JT. *Clostridium difficile* infection. N Engl J Med 2015;372:1539–1548. 25875259 10.1056/NEJMra1403772

[2] Langford BJ , Seah J , Chan A , Downing M , Johnstone J , Matukas LM. Antimicrobial stewardship in the microbiology laboratory: impact of selective susceptibility reporting on ciprofloxacin utilization and susceptibility of gram-negative isolates to ciprofloxacin in a hospital setting. J Clin Microbiol 2016;54:2343–2347. 27385708 10.1128/JCM.00950-16 PMC5005502

[3] Barlam TF , Cosgrove SE , Abbo LM , et al. implementing an antibiotic stewardship program: guidelines by the Infectious Diseases Society of America and the Society for Healthcare Epidemiology of America. Clin Infect Dis 2016;62:e51–e77. 27080992 10.1093/cid/ciw118 PMC5006285

[4] Van TT , Minejima E , Chiu CA , Butler-Wu SM. Don’t get wound up: revised fluoroquinolone breakpoints for enterobacteriaceae and pseudomonas aeruginosa. *J Clin Microbiol* 2019. doi: 10.1128/JCM.02072-18. 10.1128/JCM.02072-18 PMC6595448 31043468

[5] CLSI. Analysis and Presentation of Cumulative Antimicrobial Susceptibility Test Data, 4 *th Edition.* Wayne PA: Clinical and Laboratory Standards Institue; 2014.

[6] McNulty CA , Lasseter GM , Charlett A , et al. Does laboratory antibiotic susceptibility reporting influence primary care prescribing in urinary tract infection and other infections? J Antimicrob Chemother 2011;66:1396–1404. 21398297 10.1093/jac/dkr088

[7] Pulcini C , Tebano G , Mutters NT , et al. Selective reporting of antibiotic susceptibility test results in European countries: an ESCMID cross-sectional survey. Int J Antimicrob Agents 2017;49:162–166. 28093208 10.1016/j.ijantimicag.2016.11.014

[8] Echevarria K , Groppi J , Kelly AA , Morreale AP , Neuhauser MM , Roselle GA. Development and application of an objective staffing calculator for antimicrobial stewardship programs in the Veterans. Health Administration. Am J Health-Sys Pharm 2017;74:1785–1790. 10.2146/ajhp160825 28947624

[9] Nhan D , Lentz EJM , Steinberg M , Bell CM , Morris AM. Structure of antimicrobial stewardship programs in leading US hospitals: findings of a nationwide survey. Open Forum Infect Dis 2019;6:ofz104. 30968055 10.1093/ofid/ofz104 PMC6451647

[10] Graham M , Walker DA , Haremza E , Morris AJ. RCPAQAP audit of antimicrobial reporting in Australian and New Zealand laboratories: opportunities for laboratory contribution to antimicrobial stewardship. J Antimicrob Chemother 2019;74:251–255. 30295792 10.1093/jac/dky398

